# Alternative Macroautophagic Pathways

**DOI:** 10.1155/2012/189794

**Published:** 2012-03-27

**Authors:** Katrin Juenemann, Eric A. Reits

**Affiliations:** Department of Cell Biology and Histology, Academic Medical Center, Meibergdreef 9, 1105 AZ Amsterdam, The Netherlands

## Abstract

Macroautophagy is a bulk degradation process that mediates the clearance of long-lived proteins, aggregates, or even whole organelles. This process includes the formation of autophagosomes, double-membrane structures responsible for delivering cargo to lysosomes for degradation. Currently, other alternative autophagy pathways have been described, which are independent of macroautophagic key players like Atg5 and Beclin 1 or the lipidation of LC3. In this review, we highlight recent insights in indentifying and understanding the molecular mechanism responsible for alternative autophagic pathways.

## 1. Introduction

Autophagy, which is highly conserved from yeast to human, is a cellular degradation pathway that delivers cytoplasmic substrates to lysosomes for subsequent degradation. In contrast to the Ubiquitin-Proteasome System (UPS), which directly degrades monomeric proteins in the cytoplasm or nucleus, autophagy targets a wide spectrum of substrates including long-lived proteins, protein aggregates, and organelles towards lysosomes for subsequent degradation. In mammalian cells, autophagy occurs under basal conditions but can be stimulated by various stress conditions including starvation, hypoxia, and treatment with apoptosis-inducing compounds like rapamycin. In addition to its role in maintaining cellular homeostasis, autophagy is implicated in a wide range of physiological and pathological conditions, including early embryological development, clearance of pathogens, tumor suppression, and antigen processing and presentation [1]. In order to target cytoplasmic proteins to the lysosomes, several autophagic pathways exist, including microautophagy, chaperone-mediated autophagy (CMA), and macroautophagy. While micro- and macroautophagy can occur both in eukaryotes, plants, and fungi, CMA has only been observed in mammals. Microautophagy is the direct engulfment of cytoplasm or whole organelles by invagination or protrusion of arm-like structures of the lysosomal membrane. Here, the sequestration of cytoplasmic cargo occurs directly at the vacuole surface [2–5]. The second type of autophagy is CMA, which selectively degrades specific cytosolic proteins containing a pentapeptide motif (KFERQ) that is recognized by the heat shock cognate protein 70 (Hsc70) [6, 7]. The chaperone-substrate complex subsequently binds the lysosome through interaction with the receptor Lamp-2a on the lysosomal membrane [8]. Upon delivery by Hsc70, the substrate protein is unfolded before crossing the lysosomal membrane and lysosomal Hsc70 pulls the substrate into the lysosomal matrix where it is degraded by proteases [9]. The last but main type of autophagy is macroautophagy. Here, double-membrane vesicles, termed autophagosomes, are formed and sequester portions of cytosolic content or intact organelles (such as mitochondria) [10]. These autophagosomes are subsequently transported in a dynein-dependent manner along microtubules and fuse with endosomes or directly with lysosomes to form autolysosomes, resulting in breakdown of their contents by hydrolytic enzymes [11]. Macroautophagy is the major cellular pathway to recycle cell components including long-lived proteins and organelles, thereby providing nutrients for the eukaryotic cell, and it is activated under nutrient starvation. Additionally, macroautophagy is essential for development, cell survival, and tissue-specific processes [12, 13]. The initiation of autophagosome formation starts with the phagophore (autophagosome precursor), and recent studies indicate that the source of the membrane is the endoplasmatic reticulum (ER) [14, 15]. However, alternative sources for the autophagosomal membrane have been proposed, including the Golgi apparatus, and therefore the origin of the phagophore membrane still remains unresolved [16, 17].

## 2. Macroautophagy

Macroautophagy is a multistep process controlled by proteins termed autophagy-related (Atg) proteins [18]. The formation of the phagophore requires the class-III-phosphatidylinositol 3-kinase (PI3K) Vps34 that forms a complex with Beclin 1 (the mammalian orthologue of yeast Atg6). Inhibitors of Vps34 such as methyladenine (3-MA) or wortmannin can be used to inhibit macroautophagy since they prevent autophagosome nucleation [19–22]. The elongation of the autophagosomal membrane is dependent on two ubiquitin-like conjugation systems [23]. Atg5-Atg12 controls autophagy, where Atg12 is conjugated to Atg5 in a step that requires Atg7 (ubiquitin-activating-enzyme (E1)-like) and Atg10 (ubiquitin-conjugating-enzyme (E2)-like). The Atg5-Atg12 conjugation depends on Vps34 activity and is localized onto the phagophore where it dissociates upon formation of the autophagosome. Atg5-Atg12 forms a complex with Atg16L that modulates the next process, the ubiquitin-like conjugation of LC3-I (mammalian orthologue of Atg8). The protein LC3 is proteolytic activated by Atg4, which cleaves the C-terminus of LC3, thereby generating a cytosolic LC3-I, which subsequently conjugates with phosphatidylethanolamine (PE) to form membrane-associated LC3-II [24]. This process requires Atg7 and Atg3, and the Atg16L complex modulates the LC3-I lipidation by acting like an E3-like enzyme [25]. Although the Atg5-Atg12 conjugation dissociates upon completion of the autophagosome formation, LC3-II persists with the autophagosomal membrane even after fusion with a lysosomes and is regarded as a key marker for autophagosomes. Atg4 is also involved in the deconjugation reaction of LC3-II, as Atg4 delipidates LC3-II and removes it from the autophagosomal membrane [24, 26]. A pathway that negatively regulates macroautophagy is controlled by mTOR (mammalian target of rapamycin). mTOR activity is inhibited under starvation conditions, which activates starvation-induced macroautophagy. Recently, two new key regulators of macroautophagy, named NIX and DOR, which directly interact with the autophagosome-membrane-associated protein LC3, were identified [27]. Nix, a Bcl2-related protein localized the outer mitochondrial membrane, has a function as an adaptor protein and recruits autophagic components to mitochondria via its WXXL-like domain facing the cytoplasm [28–30]. NIX is upregulated during erythroid differentiation where a lack of mitochondria is achieved by mitophagy [27, 31, 32]. Interestingly, NIX-deficient mice show remaining mitochondria in matured red blood cells suggesting that NIX is a selective autophagy receptor that mediates mitochondrial clearance, as it directly binds LC3, but it may also target mitochondria for degradation in an LC3-independent manner [27, 33, 34]. Intriguingly, in the same issue of EMBO reports, another new autophagy-related protein was reported. Mauvezin et al. identified the nuclear cofactor of thyroid hormone receptors, termed DOR (diabetes- and obesity-regulated gene), as a new player of macroautophagy [35]. Stress-induced macroautophagy by starvation or rapamycin leads to release of DOR from the nucleus in DOR-transfected HeLa cells. This relocalization was not observed in the absence of cellular stress, indicating that cellular stress is essential to trigger DOR recruitment to the cytoplasm. DOR is associated with early autophagosomes via interaction with LC3 and GATE16 but does not colocalize with autolysosomes suggesting that DOR has a regulatory role in recruiting substrates for autophagic clearance. In addition, DOR-transfected HeLa cells show increased turnover of proteins and elevated numbers of autophagosomes compared to untreated cells. It has yet to be discovered which role DOR is playing, as it may be involved in targeting proteins to autophagy or in the formation and nucleation of the autophagosome. Whether DOR activation affects autophagy-induced alterations in cell survival remains to be established.

Macroautophagy was originally described to target intracellular organelles such as mitochondria and big protein complexes, but over the years it became clear that also most long-lived proteins are degraded via autophagic pathways. In contrast, the other main degradation machinery in the cell, the UPS, degrades mainly soluble short-lived and misfolded proteins that are targeted to the proteasome following ubiquitination (using a series of E1-E2-E3 enzymes to specifically target proteins for destruction). The proteasome is present in both the cytoplasm and the nucleus and can unfold and degrade single proteins into small peptide fragments that are subsequently recycled by peptidases. Interestingly, impairment of the proteasome leads to an increase in macroautophagy, indicating that macroautophagy can target accumulating ubiquitinated proteasomal clients when required [36–39]. In contrast, impairment of macroautophagy does not lead to increased proteasome activity. Inhibition of macroautophagy does not affect the catalytic activity of the proteasome but results in the accumulation of the macroautophagy cargo receptor p62 (also termed SQSTM1) which competes with the proteasome for ubiquitinated substrates. Indeed, silencing of p62 increases the amount of UPS clients, whereas overexpression of p62 inhibits degradation of the proteasomal substrates p53 and Ub^G76V^-GFP [40, 41]. As p62 links ubiquitinated proteins via its ubiquitin-associated (UBA) domain to the autophagic protein LC3-II and is itself degraded in the process, inhibition of macroautophagy leads to p62 accumulation which will compete and frustrate other ubiquitin-binding proteins that participate in proteasome-mediated degradation.

## 3. Alternative Autophagic Pathways

Failure of the UPS or autophagic pathways to efficiently clear proteins leads to the accumulation and subsequent aggregation of these proteins, which is a hallmark of various neurodegenerative disorders including polyglutamine (polyQ) disorders such as Huntington's disease. Here, fragments of the disease-related protein containing the polyQ tract initiate aggregation and toxicity, which can be mimicked by expressing the expanded polyQ sequence as a peptide [42]. Apparently, not all peptides are efficiently degraded by peptidases, which led to our recently published study where we examined potential alternative degradation machineries when peptidases would fail in degrading protein fragments [43]. In this study, we introduced peptidase-resistant peptides into living cells and observed a perinuclear accumulation of these peptides in time. Surprisingly, these structures did not represent aggregates or inclusion bodies as observed previously for aggregation-prone protein fragments, as no UPS components or chaperones were recruited. Although initially present in the nucleus and cytoplasm, the peptides were efficiently targeted to lysosomes within a few hours upon introduction into cells, and subsequently degraded. Our results indicate, therefore, that similar to the described increase in autophagy upon proteasome impairment, a backup mechanism exists for small protein fragments that show peptidase resistance. Intriguingly, this mechanism was very efficient for peptides of the average size of proteasomal products (6–9 amino acids), but far less for extended peptides over 25–30 amino acids which remained cytoplasmic for prolonged periods [43]. Similar to expanded polyQ peptides of disease-related lengths, these expanded peptidase-resistant peptides were more resistant to clearance by lysosomes suggesting that this pathway is particularly efficient for small peptides generated by the proteasome. It is tempting to speculate that this mechanism evolved as a backup to peptidases in the clearance of proteasome-derived peptides and emphasizes the need to identify the involved proteins. Using correlative microscopy, we mainly observed double-membrane vesicles that contained peptides and that colocalized with LC3. The colocalization increased when we used Bafilomycin A1 to impair maturation into autolysosomes. In contrast, we could prevent colocalization of LC3 with the macroautophagy inhibitor 3-MA, suggesting that the macroautophagic pathway took over the clearance of these peptides. Unexpectedly, inhibition of macroautophagy by inhibitors such as 3-MA or knockdown of Atg5 prevented recruitment of LC3 but did not affect the trafficking of these peptides into lysosomes or their subsequent degradation. Apparently, LC3 was recruited during the trafficking of peptides towards lysosomes yet was not essential. Similar to the knockdown of the various LC3 isoforms (LC3A-C), knockdown of the Atg8-related GABARAP proteins, that can interact with autophagosomes, did not affect the targeting of peptides towards lysosomes [44, 45]. As knockdown of Atg5 or WIPI-1 did not affect the trafficking and subsequent degradation of peptides in lysosomes, we concluded that these peptides entered lysosomes via a pathway different from macroautophagy. CMA is also unlikely to contribute to this pathway as the peptides lack a CMA motif and peptides composed of D-amino acids, which are unable to bind chaperones like Hsc70, were also trafficking via this pathway. Finally, we also examined endosomal microautophagy, a process that delivers soluble cytosolic material to vesicles of late endosomes or multivesicular bodies (MVBs) [46, 47]. Although accumulated peptides colocalized with internalized MHC class II molecules which may lead to so-called cross-presentation to the immune system (unpublished observation), knockdown of the sorting complexes required for transport (ESCRTs) I and III showed no effect on peptide accumulation in lysosomes. As no recruitment of ESCRT regulators towards accumulated peptides was observed, this indicates that the endosomal microautophagy pathway is not involved in the trafficking and clearance of the peptidase-resistant peptides.

The accumulation and subsequent lysosomal degradation of cytoplasmic proteins independent of known autophagy pathways have been previously observed in several studies (as described below), although in each case differences in sensitivity to autophagy inhibitors and the involvement of various Atg proteins were reported. Interestingly, in a study using Green Fluorescent Protein (GFP) like fluorophores, a pathway reminiscent of that we observed for the peptidase-resistant peptides was observed [40]. Various GFP-like fluorophores have been shown to form dimers, tetramers, or even larger complexes. Upon expression, these fluorescent proteins formed cytoplasmic fluorescent puncta that resembled lysosomes, similar as observed for the peptidase-resistant peptides [48]. However, the accumulating fluorophore proteins including monomeric RFP1 (mRFP1) showed resistance to lysosomal degradation and retain fluorescence, in contrast to the peptides. Trafficking of the GFP-like proteins and the peptidase-resistant peptides was not affected in Atg5-deficient mouse embryonic fibroblasts, suggesting that they may be targeted to lysosomes by a similar pathway (although no other macroautophagy markers were examined for the fluorescent proteins). So is the constitutive macroautophagy-independent targeting of cytoplasmic proteins and peptides to autolysosomes restricted to introduced peptides and GFP-like fluorophores?

At least two alternative autophagy pathways have been described: an Atg5/Atg7-independent pathway and the so-called noncanonical autophagy pathway, which is independent of Beclin 1 (Table 1). The Atg5/Atg7-independent autophagic pathway was recently discovered in mouse embryonic fibroblasts (MEF) lacking Atg5 and Atg7 that were treated with the cytotoxic stressor etoposide, which caused an equivalent appearance of autophagic vacuoles when compared to wild-type cells [49]. Moreover, autophagic vacuoles were also found in starved Atg5-/- cells. The Atg5/Atg7-independent form of autophagy does not involve the lipidated conjugate LC3-II, which is membrane associated. Interestingly, equivalent numbers of LC3-positive and LC3-negative autophagosomes were observed in etoposide-treated wild-type cells, suggesting that conventional and alternative autophagic pathway occur at the same time. The proteins Atg5, Atg7, and LC3, which are important in the ubiquitin-like conjugation system for the autophagosome elongation, are not involved in this alternative form of autophagy. However, silencing of Beclin 1 and Vps34 decreased the amount of autophagosomes, indicating that the PI3K complex, which acts upstream of initiation of autophagosome formation, is still required in etoposide- or starvation-induced autophagy in Atg5-/- cells. Accordingly, protein degradation via this pathway was inhibited by the PI3K inhibitor 3-MA. Furthermore, silencing of components of the Ulk1 complex, a mammalian serine/threonine protein kinase that plays a key role in the initial stages of autophagy, decreased autophagic vacuoles, suggesting that the Ulk1 complex is needed for Atg5/Atg7-independent autophagy [49].

Apoptosis-induced stress, for example, by staurosporine, resveratrol, or H_2_O_2_ can also induce the so-called non-canonical autophagy pathway, where autophagosomes can be formed independent of Beclin 1 or Vps34 and with an insensitivity to 3-MA [50–52]. However, this specific pathway still requires Atg7-activity for LC3-I lipidation and is, therefore, different from the Atg5/Atg7-independent pathway described above [49]. Furthermore, Scarlatti et al. have shown that resveratrol inhibits the mTOR activation by a direct inhibitory effect on the upstream class 1A PI3K [50]. Similarly, a Beclin 1-independent pathway has been reported in neuronal cells treated with the neurotoxin 1-methyl-4-phenylpyridinium (MPP+) [53] and in other cellular systems in response to various drugs [54, 55]. These studies have shown that several agents stimulate autophagic cell death through Beclin 1 in canonical autophagy pathways [56]. Recently, evidence emerged that autophagy and cell death are induced independent of Beclin 1 and Vps34. In breast cancer cells, resveratrol induces autophagic cell death in a Beclin 1-independent manner [50]. Silencing of Atg7 impairs the cellular death elicited by resveratrol. In dopaminergic neuronal cells, the neutotoxin MPP+ induces Beclin 1-independent autophagy and cell death [53]. As most studies on the noncanonical pathway used compounds to induce cell death, it is tempting to link the noncanonical autophagy pathway to a death execution mechanism or cell survival. However, it has also been suggested that the independency of the noncanonical autophagy pathway may provide an adaptation to loss of Beclin 1, for example, in various tumors where Beclin 1 is deleted, in immune cell development, and may even be an evolutionary way to circumvent inhibition of Beclin 1 by various viruses in order to prevent autophagy [57–59].

None of these alternative autophagy pathways seem to correspond to the trafficking we observed for the peptidase-resistant peptides, as the Atg5/Atg7-independent pathway is still 3-MA sensitive (in contrast to the peptide targeted to lysosomes), while the noncanonical pathway (Beclin 1-independent) is 3-MA insensitive but still depends on LC3. Thus, lysosomal degradation of peptidase-resistant peptides and proteins, as we and others have demonstrated [35, 41–45], defines a novel authophagy route independent of known regulators of the constitutive macroautophagic pathway like Beclin 1, Atg5 or LC3. A better understanding of the role of these alternative autophagic pathways and their molecular regulators raise to two crucial questions: (1) What is the origin of the autophagic membrane in the different autophagic routes, and (2) Which stimuli trigger the different autophagic pathways?

In mammalian macroautophagy, various sources for the origin of the autophagosome membrane have been proposed including the ER, the Golgi complex, the plasma membrane, and the mitochondria [17, 60–66]. Alternatively, *de novo* synthesis of a nucleating structure, the phagophore, is proposed to elongate by the addition of lipids via the integral membrane protein Atg9 [67–70]. Atg9 seems to be a key regulator in regulating the formation and expansion of nascent autophagosomes. Unfortunately, the identity of proteins that partition to the autophagosomal membrane remains largely unknown. Therefore, attempting to determine the origin of the autophagosomal membrane based on the associated proteins remains a challenge [71]. Alternatively, others attempted to determine the source of the autophagosomal membrane by inspecting its thickness and lipid composition [15]. Several studies reported that the autophagosomal membrane can be classified as of a thin type (6–8 nm), similar to membranes of the ER and mitochondria [60, 72–75]. Furthermore, lipid structures enriched in PI3P (known as omegasomes) were formed in the vicinity of ER membranes after amino acid starvation, suggesting that these omegasomes originate from the ER [76–79]. As the omegasomes carry autophagosomal proteins like Atg5 and LC3, they may represent the source of isolated membranes required for autophagosome expansion. In contrast, in the Atg5/Atg7-independent autophagic pathway, autophagosomes with membranes of the thick type (9-10 nm) were observed, similar to membranes of lysosomes and the *trans*-Golgi network [49]. Intriguingly, unlike the conventional pathway the alternative Atg5/Atg7-independent form of autophagy is blocked by brefeldin A, indicating that autophagosomes are derived from the Golgi-apparatus. Etoposide-induced Atg5/Atg7-independent autophagy is accompanied by colocalization of markers of the *trans*-Golgi and late endosomes (such as the mannose 6-phosphate receptor, TGN38, and Rab9) with Lamp-2-positive autolysosomes, further pointing to the requirement of the *trans*-Golgi or late endosomes in this alternative form of autophagy. Indeed, silencing of Rab9 or expression of a Rab9 dominant negative mutant established an essential role for Rab9 in membrane expansion from isolated membranes and led to an accumulation of isolated membranes after silencing of Rab9 but not upon inhibition of Ulk1 or Beclin 1. Since the Atg5/Atg7-independent type of alternative autophagy is activated by starvation and the stress-inducing reagent etoposide, but not by rapamycin, this suggests that a specific stimulus for induction of autophagy activates nonconventional macroautophagy with different lipid structures compared to conventional macroautophagy. To the best of our knowledge, there is no clear data on the source of membrane for the Beclin 1-independent noncanonical autophagy pathway.

**Figure 1:**
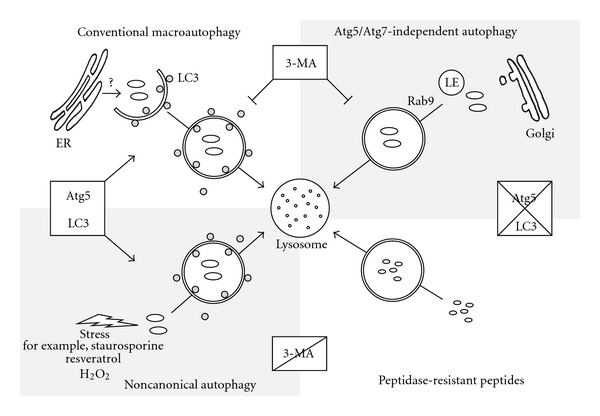
Alternative macroautophagic pathways lead to lysosomal degradation. At least four autophagic pathways can be distinguished that all show double-membrane autophagic structures and end in lysosomal degradation of cytoplasmic cargo. Conventional macroautophagy is hallmarked by the recruitment of lipidated LC3 to autophagosomal membranes that may origin from the endoplasmatic reticulum (ER). This process is dependent on Atg5 and Beclin 1 and can be inhibited by 3-methyladenine (3-MA). In contrast, the observed Atg5/Atg7-independent autophagy pathway forms Rab9-positive double-membrane vesicles derived from the *trans*-Golgi network and late endosomes (LE), and while it can be inhibited by 3-MA and is dependent on Beclin 1, the process is independent of Atg5 and LC3. Almost similar, the degradation of accumulated peptidase-resistant peptides is independent of Atg5 and LC3 and is also insensitive to 3-MA treatment. Finally, the noncanonical autophagy pathway induced by different stress factors is dependent on Atg5 and LC3 and independent of Beclin 1 but cannot be impaired by 3-MA.

So far, several sources have been proposed to provide the putative moiety of autophagosomal membranes. However, autophagosomal membranes could derive from multiple membrane sources and the origin of lipids may vary dependent on the cell type, the stimulus that triggers the degradation, and the type of cargo for autophagic destruction (proteins, aggregates or even whole organelles). As shown in Figure 1, there are now at least three alternative pathways that target cytosolic content to lysosomes, which can be discriminated by their dependence on Atg5 and 3-MA (Figure 1). The identification of key players and the origin of membrane structures involved in alternative autophagic pathways will be important for the understanding of molecular mechanism regulating these various types of autophagy.

## Figures and Tables

**Table 1:** Types of alternative macroautophagic pathways.

Alternative macroautophagic pathways	Macroautopagic molecules involved	Macroautopagic molecules not involved	Induction	Cell type	Reference
Beclin 1-independent	Atg5 Atg7 Ulk1/2 LC3	Beclin 1 (Vps34)	Resveratrol	MCF-7 (breast cancer cells)	[50]
			Staurosporine Etoposide MK801	primary cortical neurons	[51]
			H_2_O_2_	RAW 264.7 (macrophage cells)	[52]
			MPP+	SH-SY5Y (neuroblastoma cells)	[53]
				primary dopaminergic neurons	
			As_2_O_3_	ovarian cells	[55]

Atg5/Atg7-independent	Beclin 1 Vps34 Ulk1 Fip200	Atg5 Atg7 Atg9 Atg12 Atg16 LC3	Etoposide Staurosporine Starvation	Atg5-/- MEF Atg7-/- MEF wt MEF	[49]

Degradation of peptidase-resistant peptides	LC3 (but not essential)	Atg5 WIPI-1 p62 Tsg101 Vps24	Resistance against cytoplasmic peptidases	HeLa Atg5-/- MEF wt MEF	[43]
